# Neuropsychiatric Symptoms in Parkinson’s Disease Patients Are Associated with Reduced Health-Related Quality of Life and Increased Caregiver Burden

**DOI:** 10.3390/brainsci12010089

**Published:** 2022-01-11

**Authors:** Hannah von Eichel, Johanne Heine, Florian Wegner, Sophia Rogozinski, Stephanie Stiel, Adrian Groh, Lea Krey, Günter U. Höglinger, Martin Klietz

**Affiliations:** 1 Department of Neurology, Hannover Medical School, Carl-Neuberg-Straße 1, 30625 Hannover, Germany; Hannah.C.V.Eichel@stud.mh-hannover.de (H.v.E.); Heine.Johanne@mh-hannover.de (J.H.); Wegner.Florian@mh-hannover.de (F.W.); Rogozinski.Sophia@mh-hannover.de (S.R.); Krey.Lea@mh-hannover.de (L.K.); Hoeglinger.Guenter@mh-hannover.de (G.U.H.); 2 Institute of General Practice, Hannover Medical School, Carl-Neurberg-Straße 1, 30625 Hannover, Germany; Stiel.Stephanie@mh-hannover.de; 3 Department of Psychiatry, Social Psychiatry and Psychotherapy, Hannover Medical School, Carl-Neuberg-Straße 1, 30625 Hannover, Germany; Groh.Adrian@mh-hannover.de

**Keywords:** Parkinson’s disease, neuropsychiatric symptoms, SEND-PD, caregiver burden, health-related quality of life, depression

## Abstract

Parkinson’s disease (PD) is a progressive neurodegenerative disorder resulting in reduced health-related quality of life (HR-QoL) of people with PD (PwP) and their caregivers. Furthermore, there is an accumulating burden on caregivers of patients in advanced stages of the disease. In previous studies, motor- and non-motor-symptoms of PwP have been identified to contribute to reduced HR-QoL and an increased caregiver burden. This cross-sectional observational study aimed to study the influence of neuropsychiatric symptoms measured with the Scale for Evaluation of Neuropsychiatric Disorders in Parkinson’s Disease (SEND-PD) questionnaire on the HR-QoL of PwP, as well as the caregiver burden. Analyses revealed a significant association between SEND-PD subscale mood/apathy and reduced HR-QoL in PwP, measured by the Parkinson’s disease quality of life questionnaire (PDQ-8) (*p* < 0.001). Furthermore, mood/apathy was significantly correlated with caregiver burden (*p* = 0.001) in the multiple linear regression analysis. Hence, neuropsychiatric symptoms were found to have a profound impact on the HR-QoL of PwP, as well as on caregiver burden. Since neuropsychiatric symptoms were one of the main predictors for caregiver burden, physicians of PwP should treat these symptoms to stabilize caregiver burden, as well as HR-QoL in PwP and their caregivers.

## 1. Introduction

Parkinson’s disease (PD) is a neurodegenerative movement disorder with progressive restrictions in health-related quality of life (HR-QoL) and loss of patient’s autonomy in advanced stages [1]. As a result, mostly family members, and especially spouses undertake the care of the patient and become the informal caregiver. However, in several cases, additional professional caregivers or institutionalization of the patient are inevitable [2,3].

In Germany, the prevalence of PD is about 0.5% of the general population. However, prevalence and incidence are increasing with higher age [4]. People with PD (PwP) suffer from the classical motor symptoms of tremor at rest, bradykinesia, rigidity, and postural instability. Interestingly, James Parkinson already observed non-motor symptoms like cognitive decline, anxiety, depressive mood, and hallucinations in the famous “essay on the shaking palsy” [5]. In recent years, the impact of non-motor symptoms on HR-QoL was established. In the early phase, PwP often suffer from distinct cognitive impairments, anxious mood, and depressive symptoms [6]. In the late stages, PwP suffer from more severe neuropsychiatric symptoms like hallucinations, depression, and dementia [7]. Several hypothesizes have been developed, inter alia by molecular imaging methods, to explain the pathophysiology of non-motor symptoms and neuropsychiatric symptoms. Neuropsychiatric symptoms, particularly mood and depression, seem to be associated with a decreased monoamine uptake in the thalamus in PwP. Additionally, reduced dopamine transporter uptake in other brain regions like the caudate nucleus, ventral striatum, and putamen appear to play a role. Anxiety was found to be associated with reduced caudate dopamine transporter uptake [8,9,10,11,12,13]. Furthermore, a relationship between apathy and the reduction of dopaminergic neurons in the striatum has recently been discussed [9,14]. For instance, a previous study using dopamine transporter (DAT) single photon emission computed tomography (SPECT) found lower DAT levels in PwP with apathy compared to PwP without apathy in the right caudate [15]. In contrast, another study did not find a significant relationship between DAT binding in striatal sub-regions and apathy in PwP [16]. Hence, the role of dopaminergic neurons in the pathophysiology of apathy remains ambiguous. Aside from dopamine, serotonin is suspected to play a role in the pathophysiology of neuropsychiatric symptoms in PD [17].

Neuropsychiatric symptoms impact on both the patient’s HR-QoL and caregiver burden in PD and other neurodegenerative diseases [18,19,20,21]. For instance, neuropsychiatric symptoms were identified to lead to higher caregiver burden and reduced HR-QoL in caregivers taking care of people with Alzheimer’s Disease [21]. In Huntington’s Disease, depressive symptoms of the patient along with apathy are predictors for reduced HR-QoL and increased caregiver burden, as reported in the past [22,23].

In PD, neuropsychiatric symptoms like depression and anxiety, assessed by the Hospital Depression and Anxiety Scale (HADS) in a former study, also showed a significant influence on the HR-QoL of PwP [24]. Furthermore, in previous studies, neuropsychiatric symptoms measured by the newly validated Scale for Evaluation of Neuropsychiatric Disorders in Parkinson’s Disease (SEND-PD) in a Mexican cohort, particularly mood/apathy and psychosis, could be identified to contribute to caregiver burden [18,25]. The recently developed questionnaire SEND-PD offers the possibility to quickly assess neuropsychiatric symptoms in PD patients, and was validated in a German cohort not long ago [26,27].

Until now, there is no data on the impact of neuropsychiatric symptoms measured with the SEND-PD on HR-QoL of PwP and caregiver burden in a German cohort.

The aim of the present study was to analyze the impact of neuropsychiatric symptoms determined by the SEND-PD on patients’ and caregivers’ HR-QoL as well as the burden of the informal caregiver measured by the disease-specific PD caregiver burden questionnaire (PDCB).

## 2. Materials and Methods

### 2.1. Participants

Ethical approval for this study was granted by the local Ethics Committee of Hannover Medical School (No. 3178-2016, Amendment in 2018). The research was conducted in accordance with the World Medical Association Declaration of Helsinki. Participants were recruited via the local outpatient department, the neurological ward, and from German patient groups. The questionnaires were sent via mail to the participants. They received written information and gave their informed consent before inclusion. A total of 128 patient/caregiver couples were contacted to participate in this study, and 89 patients and 84 caregivers returned suitable datasets for statistical analysis, as well as their written informed content. Only patients with PD and a disease duration of at least one year since the onset of motor-symptoms, who had been diagnosed by a neurologist and met the Movement Disorders Society (MDS) diagnosis criteria for PD [28], were able to participate in the study.

Patients in institutional care were not included in our study, and patients with atypical Parkinsonism or without any informal caregiver (e.g., spouses or children) were excluded from this study. Only the person serving as the primary caregiver who spent most (caregiving) time with the patient was considered in this study. Professional caregivers were excluded from this study. 

No participant received any financial compensation for study inclusion. Completion of the study questionnaire took about 30 min for PwP and their informal caregivers, respectively.

### 2.2. Measures

Patients rated their Hoehn and Yahr stage at the best clinical stage to classify their motor impairment, as was also done in other studies of our group [29,30,31]. It reached a minimum of one point (unilateral symptoms) to a maximum of five points (confinement to bed or wheelchair because of PD) [32].

The “Parkinson’s disease quality of life questionnaire 8” (PDQ-8) is a questionnaire to assess patients’ functioning and well-being in eight different domains and is used to determine the patients’ HR-QoL [33]. The eight questions on a five-point Likert scale reach from a minimum of 0 to a maximum of 4 points. A higher total score of the questionnaire is associated with a lower HR-QoL of the patient. This total score, which reaches 0 to 32 points, is converted as a percentage that represents the HR-QoL restrictions. A high percentage indicates severe HR-QoL restrictions of the PwP. We asked caregivers to support patients with possible disease-related impairment in completing the PDQ-8 to ensure correct answers and avoid anosognosia (as also done in [34,35,36]).

Furthermore, patients answered the “Movement Disorders Society Unified Parkinson’s disease rating scale” (MDS-UPDRS) part II to scale their impairment of daily living regarding motor impairments in PD. It consists of 13 items on a five-point Likert scale from 0 (no symptoms) to 4 (severe symptoms) each, with a maximum of 52 points indicating the worst level of symptoms [37].

“Beck’s depression inventory” (BDI) was used to assess patients’ and caregivers’ depressive symptoms. The questionnaire ranges from 0 to 63 points: 0–13 no depression, 14–19 mild depression, 20–28 moderate depression, and 29–63 severe depression [38].

One main goal of this study was to evaluate neuropsychiatric symptoms by the “Scale for Evaluation of Neuropsychiatric Disorders in Parkinson’s Disease” (SEND-PD [26]). It consists of 12 items divided into three subscales: psychotic symptoms (4 items), mood/apathy (5 items) and disturbance of impulse control (3 items). For each subscale, the items are scored from 0 (not present) to 4 (very severe) and a total score for each subscale is calculated, with a maximum of 16 for psychotic symptoms, 20 for mood/apathy, and 12 for disturbance of impulse control. We assumed a neuropsychiatric condition to be present at a score of 1 or higher [26].

Patients and caregivers were asked to provide some general demographic information, including age and disease duration in years at time of the survey. Caregivers also estimated their caregiving hours per day. 

To evaluate caregiver burden, we used the validated “German version of the Parkinson’s disease caregiver burden questionnaire” (PDCB) [36,39]. The questionnaire contains 20 items on a five-point Likert scale, with each item reaching from 0 points minimum to 4 points maximum. Caregivers can score a maximum of 80 points. In the second part of the questionnaire, participants value their global burden as a caregiver on a scale from 0 to 100. To calculate the total caregiver burden score, 20% of the global burden is added to the sum of the questionnaires first part. The total PDCB can range from 0 to 100, with higher scores indicating higher caregiver burden.

Moreover, the “short-form 36 health survey” (SF-36) evaluates the health-related quality of life (HR-QoL) of the caregivers in eight categories [40,41]. The subscale scores of each category are expressed as a percentage. A value of 0 indicates maximum impairment, whereas 100 displays no reported impairment. To take the high correlation between the categories into consideration, an average value over these eight subscale scores was calculated as also done in previous studies of our group [31].

### 2.3. Analyses

The collected data were analyzed by using the Statistical Package for the Social Sciences version 25.0 (SPSS, IBM, Armonk, NY, USA). Descriptive analyses are presented in mean and standard deviation or median, as well as minimum and maximum. Numbers of PwP and caregivers vary since some of the caregivers denied the participation in this study. Furthermore, numbers of PwP and caregivers differ between analysis due to the fact that some PwP and caregivers only replied to some of the questionnaires. However, we decided to include these PwP and caregivers in our study to prevent further reduction of participants. To explore the relationship between HR-QoL of PwP and other variables (disease duration, Hoehn and Yahr stage, SEND-PD psychotic symptoms, SEND-PD mood/apathy, SEND-PD impulse control disorders, MDS-UPDRS II), Spearman’s correlation coefficients were determined. Furthermore, Spearman’s correlation coefficients were calculated to investigate the relationship between caregiver burden and other disease-related variables (disease duration, Hoehn and Yahr stage, SEND-PD psychotic symptoms, SEND-PD mood/apathy, SEND-PD impulse control disorders, MDS-UPDRS II, caregiving hours, and BDI score of the caregiver). A correlation coefficient between 0.00 to 0.29 was considered as a negligible correlation, between 0.30 to 0.49 as low, between 0.50 to 0.69 as moderate, between 0.70 to 0.89 as high positive, and between 0.90 to 1.00 as a very high correlation [42]. To further analyze the relationship of neuropsychiatric symptoms and HR-QoL and caregiver burden, respectively, multiple regression analyses were performed. PDQ and PDCB, respectively, were considered the dependent variables, and the SEND-PD subscales were considered as independent variables/predictors. Due to the only moderate number of PwP and caregivers, no other predictors were included in the multiple linear regression analysis. Linear regression assumptions were checked by the inspection of graphics and met. For homoskedaticity, an additional Breusch-Pagan-Test was performed. The level of significance was set to α = 0.05/n (n = number of analyzed predictors) to correct for multiple testing.

## 3. Results

### 3.1. Patient and Caregiver Characteristics

Demographic and clinical characteristics of PwP (n = 89, 34 females) and their caregivers (n = 84, 54 females) are displayed in Table 1. Caregivers were PwP’s spouses (n = 79), children (n = 4) and children in law (n = 1). The average age of the patients was 68.7 ± 10.1 years and their disease duration at the time of the survey was 10.7 ± 6.6 years. As characterized by the Hoehn and Yahr stage, PwP were in moderate to advanced stages of PD with a median of 3 points. HR-QoL, measured by PDQ-8, was on average at 34.8 ± 17.8, while the patients’ motor-impairment of daily living (UPDRS part II) was at 17.7 ± 10.6 points. The individual subscales of SEND-PD displayed the following values: psychotic symptoms 1.3 ± 2.2, mood 4.9 ± 4.4 and impulse control 1.1 ± 1.5. Psychotic symptoms were reported by 49%, whereas 87% showed alterations in mood/apathy and 54% presented with impulse control disorders, respectively. More detailed information regarding the neuropsychiatric symptoms depending on disease duration and PwP’s age can be found in the Appendix A (Table A1 and Table A2).

Primary caregivers were, on average, 66.2 ± 10.6 years of age. They reported a mean of 5.5 ± 6.5 caregiving hours per day. Overall, caregiver burden, measured by the PDCB, was moderate, with a mean score of 31.5 ± 16. On the BDI, caregivers reached an average of 9.2 ± 6.8 points. The HR-QoL, estimated by the SF-36 total, was moderate with 64 ± 19.6 points. 

### 3.2. Influence of Neuropsychiatric Symptoms on PwP’s HR-QoL

Correlation analyses regarding HR-QoL in PwP are shown in Table 2. PwP’s HR-QoL was negatively associated with the Hoehn and Yahr stage (low correlation) as well as MDS-UPDRS II (low correlation). Moreover, all dimensions of the SEND-PD correlated with PwP’s HR-QoL. The subscale psychotic symptoms and impulse control disorders displayed a low correlation, whereas the subscale mood/apathy correlation was moderate with PwP’s HR-QoL. These correlations still hold after correction for multiple testing.

To further investigate the relationship between the neuropsychiatric symptoms and PwP’s HR-QoL, multiple linear regression analysis with SEND-PD subscales as independent variables and HR-QoL represented by PDQ-8 were performed (Table 3). A significant association between SEND-PD mood/apathy and PwP’s HR-QoL could be found. To specify, higher scores in the SEND-PD mood/apathy were significantly associated with reduced HR-QoL. This association still holds after correction for multiple testing. The subscales psychotic symptoms and impulse control disorders of the SEND-PD did not show a significant correlation with PwP’s HR-QoL in multiple linear regression analysis. 

### 3.3. Influence of Neuropsychiatric Symptoms of PwP on Caregiver Burden

In a second analysis, the relationship between caregiver burden, represented by the PDCB total score, and neuropsychiatric symptoms (SEND-PD), as well as other disease-related factors was investigated (Table 4). Caregiver burden was significantly associated with MDS-UPDRS II (low correlation), caregiving hours per day (low correlation), and caregivers’ BDI (moderate correlation). Furthermore, all SEND-PD subscales correlated significantly with caregiver burden. To specify, for the subscales’ psychotic symptoms and impulse control disorders, a low correlation could be identified, whereas the subscale of psychotic symptoms showed a moderate correlation with caregiver burden. These correlations still held after correction for multiple testing. 

Further investigations regarding the relationship between neuropsychiatric symptoms and caregiver burden revealed a significant association between the SEND-PD subscale of mood/apathy and caregiver burden in multiple linear regression analysis. In fact, higher scores in SEND-PD mood/apathy were significantly associated with higher caregiver burden. In contrast, psychotic symptoms and impulse control disorders measured by the SEND-PD did not show a significant association with caregiver burden (Table 5). Further linear regression analyses did not show an association between neuropsychiatric symptoms of PwP and the caregiver burden when adding the caregiver’s gender as a covariate (data not shown). 

## 4. Discussion

In this cross-sectional study, neuropsychiatric symptoms correlated with the HR-QoL of PwP and caregiver burden. However, in the multiple linear regression analysis, only the subdimension mood/apathy showed a significant association with reduced PwP’s HR-QoL and higher caregiver burden. Furthermore, in this study, PwP’s HR-QoL showed a significant correlation with the Hoehn and Yahr stage, as well as impairment of daily living regarding motor impairments, measured by the MDS-UPDRS II. Additionally, MDS-UPDRS II displayed a significant correlation with caregiver burden. Caregiver burden was further associated with caregiving hours per day and the severity of depressive symptoms in caregivers, as represented by BDI.

### 4.1. Impact of Neuropsychiatric Symptoms on PwP’s HR-QoL

Neuropsychiatric symptoms and their impact on PwP’s HR-QoL have been increasingly brought into focus in recent years. However, assessments of neuropsychiatric symptoms were mostly either not combined in one instrument or were non-specific for PD [18]. Neuropsychiatric symptoms of PwP were mainly assessed by the Hamilton Anxiety Rating Scale (HAM-A), Montgomery–Asberg Depression Rating Scale (MADRS), and MDS-UPDRS Part I, among others [43,44,45,46,47]. The “Ardouin Scale of Behavior in Parkinson’s Disease” (ASBPD) intends to evaluate all mood and behavioral disorders in PD with one single instrument and has been reported to be a reliable and valid questionnaire to assess neuropsychiatric symptoms in PwP without dementia in people from France, Spain, the United Kingdom, and the United States [48]. However, the assessment of the PwP’s cognitive state in our cohort was not possible due to the design of the study, and a German version and validation of the ASBPD in a German cohort was not published when our study started. With the recently established and validated SEND-PD, there is now an instrument available which contains at least three features of neuropsychiatric symptoms, that is, psychotic symptoms, mood/apathy, and impulse control disorders in one instrument [27]. Although the SEND-PD has recently been validated in a German cohort, no study has been published focusing on neuropsychiatric symptoms, assessed by the SEND-PD, and HR-QoL, measured with a PD-specific scale, in PwP in a German cohort so far. Therefore, we investigated the relationship of neuropsychiatric symptoms, measured with the SEND-PD, and the PD-specific PDQ-8 representing PwP’s HR-QoL.

Neuropsychiatric symptoms were frequent in our cohort, with 88% of PwP reporting at least one neuropsychiatric symptom. This is in line with a study by Alvarado-Bolaños et al., which investigated neuropsychiatric symptoms in a Mexican cohort assessed by the SEND-PD, and detected 84% of PwP to suffer from neuropsychiatric symptoms [18].

In this study, neuropsychiatric symptoms showed a significant low to moderate correlation with PwP’s HR-QoL. However, in the multiple linear regression analysis, only the subscale of mood/apathy could be identified to be significantly associated with HR-QoL. Likewise, Alvarado-Bolaños found an association between the SEND-PD subscale of mood/apathy and HR-QoL. Depression is a very common comorbidity in PD (35–50%) and occurs already in an early stage leading to anhedonia and generally decreased interest [43,44,45]. Apathy in PwP results in general inactivity, weakness, and loss of function [49]. These signs could be misunderstood by their caregivers as laziness, ignorance, or arrogance, that might lead to arguments and therefore contribute to a decreased HR-QoL [45]. However, the relationship of mood, apathy, and depression with HR-QoL has to be interpreted with caution, since there is some overlap between the assessment instruments of HR-QoL and neuropsychiatric symptoms, as already reported in former studies [18,50].

In contrast to former studies, our cohort did not display a significant association between impulse control disorders and HR-QoL [18]. One explanation might be the use of a different analytical approach, that is, using multiple linear regression analysis in this study versus more explorative analysis, like stepwise or sequential multiple linear regression analysis in other studies [9,45]. Furthermore, in our study, only 51% of PwP showed impulse control disorders and 77% reported either no or only a mild impairment by impulse control disorders with no need of specific intervention, indicated by a score of 0 or 1 in the SEND-PD impulse control disorder subscale. Therefore, PwP and severe impulse control disorders, as well as those with more than one symptom may be underrepresented in our cohort. However, surprisingly, the proportion of PwP and impulse control disorders was higher in this cohort compared to previous studies using the SEND-PD [18,25]. Yet, those studies had a larger number of participants, and the proportion of PwP and with impulsive control disorders in our cohort was similar to a Norwegian cohort with a comparable sample size [51].

Psychotic symptoms did not show a significant association in multiple linear regression analysis. This is in line with findings by Alvarado-Bolaños et al. [18]. Similar to impulse control disorders, 77% of PwP in our cohort reported no or only a mild impairment by psychotic symptoms with no need of specific intervention, which might be a reason for the lack of significant association between psychotic symptoms and PwP’s HR-QoL. Moreover, although the SEND-PD is a valid screening method for neuropsychiatric symptoms in PwP, it does not encompass the full spectrum of psychotic symptoms, and therefore might miss PwP and different psychotic symptoms. 

However, this study showed that neuropsychiatric symptoms in total are very frequent. Hommel et al. also reported that a high prevalence of neuropsychiatric symptoms (particularly apathy, depression, and anxiety) is the strongest predictor for more neuropsychiatric symptoms in late-stage PD [52]. Hence, screening for other psychiatric comorbidities, such as with the SEND-PD, is already crucial in the early stage of the disease [52]. Moreover, assessing neuropsychiatric symptoms and mild cognitive impairment is important, as highlighted by Hanganu et al., as they are intercorrelated and may potentially predict the further clinical evolution of PD symptoms, and in particular, the patients’ quality of life [53]. A meta-analysis from Baiano et al. showed that mild cognitive impairment in PD was very common and should be detected as early as possible, since it has been identified as a risk factor for the development of dementia. Mild cognitive impairment in PD is also associated with poorer QoL, more apathy, and depressive symptoms [54]. Petkus et al. revealed that anxiety, depressive symptoms, and apathy are associated with worse executive functioning in PD [55]. This association is even higher in patients suffering additionally from mild cognitive impairment. However, due to the design of the present study, it was not possible to investigate the cognitive state of PwP by a cognitive screening questionnaire [30,51]. This issue must be addressed in future studies.

Even though there is a growing field of studies investigating the neuropathological mechanisms and clinical impact of neuropsychiatric symptoms in PD [55,56], studies analyzing this aspect have still been quite underrepresented compared to research about motor-symptoms in PD [35]. Focusing more on neuropsychiatric symptoms is important not only for PD, but also in other neurodegenerative diseases, such as Huntington’s Disease (HD) [57], and even in dystonia [26,52] to implement therapeutical interventions. For instance, a recent longitudinal study from Abbes et al. showed that subthalamic deep brain stimulation is a possible treatment in PD with neuropsychiatric symptoms. In their study, a significant improvement of neuropsychiatric fluctuations and impulse control disorders in PD over time could be detected after deep brain stimulation [58]. On the other hand, DBS in the subthalamic nucleus may increase apathy and depressive symptoms, leading to reduced HR-QoL in PwP and increased caregiver burden [59,60,61]. Therefore, considering DBS as a therapeutical option in PD with neuropsychiatric symptoms should weigh the possible advantages and disadvantages of this intervention.

### 4.2. Impact of Neuropsychiatric Symptoms of PwP on Caregiver Burden

Neuropsychiatric symptoms in PwP have also been reported to impact caregivers’ well-being. Furthermore, the presence of neuropsychiatric symptoms, especially depression, hallucinations, and psychosis, increase the risk of patients’ institutionalization and hospitalization, since caregivers feel they might not be able to take care of the patient at home [19,44,62,63]. Therefore, detecting neuropsychiatric symptoms early is important to counteract caregiver burden and transition to institutional care of the PwP. To the best of our knowledge, this is the first study in a German cohort using the SEND-PD to assess neuropsychiatric symptoms and the relationship with caregiver burden measured by the PD-specific PDCB.

In a previous study, Martinez-Martin et al. found depression and anxiety to be the most common neuropsychiatric symptom in PwP, whereas in PD with dementia, the most present symptom was apathy [25]. Moreover, Martinez-Martin et al. reported a higher caregiver burden in caregivers of PwP with neuropsychiatric symptoms compared to caregivers of PwP without neuropsychiatric symptoms measured by the SEND-PD and the Zarit Caregiver Burden Inventory. In caregivers of PwP with additional dementia, the burden was higher than in those without indicating an interdependent effect of dementia and other neuropsychiatric symptoms on caregiver burden. In both caregiver groups (caregivers of PwP with dementia as well as of PwP with normal cognition), caregiver burden was significantly associated with higher SEND-PD psychosis and mood/apathy scores [25]. Consistent with this, in our cohort, higher caregiver burden measured by the disease-specific PDCB significantly correlated with higher SEND-PD mood/apathy scores, although in the present study, it was not possible to differentiate between PwP with and without dementia due to the design of the study. One explanation for the relationship between mood alterations and caregiver burden might be the expanded need for assistance in daily activities for PwP with neuropsychiatric symptoms. For instance, due to apathy and anxiety, PwP with neuropsychiatric symptoms may develop a reduced willingness to leave the house and run errands [45]. Therefore, caregivers may take over these responsibilities in addition to their further obligations, resulting in less time for themselves. Indeed, a recent study in an American cohort determined severity of anxiety and apathy among others to be the leading contributor to the time-dependency domain in caregiver burden because of associated disability [64]. Moreover, social isolation of PwP and caregivers may occur due to apathy accompanied by stigmatization contributing to higher caregiver burden. Additionally, alterations in mood and depressive symptoms may cause fewer meaningful conversations between PwP and caregivers, resulting in a feeling of emotional distance and reduced intimacy, especially in caregivers of PwP who suffer from dementia [65]. In line with this, a recent study found more severe neuropsychiatric symptoms to be associated with decreased relationship satisfaction in PwP and spousal caregivers, which may consequently result in higher caregiver burden [66,67]. However, due to the design of this study, no causal conclusions can be drawn, and these theories have to be addressed in future studies. 

There are striking reports regarding the questions whether impulse control disorders significantly contribute to caregiver burden. One explanation might be the use of different scales to evaluate impulse control disorders and caregiver burden, as well as different statistical analysis and covariates [25,51]. In this study, no association between impulse control disorders and caregiver burden could be detected using the disease-specific SEND-PD and PDCB. However, these results need to be interpreted with caution due to the high number of PwP with no impulse control disorder or very low impairment. 

Contrary to previous studies, psychotic symptoms were not significantly associated with higher caregiver burden in multiple linear regression analysis in this study [25,68]. However, these studies used different scales to assess psychotic symptoms [68] and caregiver burden, respectively. Furthermore, different statistical analyses might have an influence on the detection of relationships between psychotic symptoms and caregiver burden [25,68]. 

In this study, screening analysis in this study showed a significant low to moderate correlation of MDS-UPDRS III, caregiver’s BDI, and caregiving hours. Moreover, the disease duration and Hoehn and Yahr stage did not correlate with caregiver burden. Due to the limited number of caregivers, we did not include these factors in the multiple linear regression analysis. However, in line with the results of this study, previous evaluations have documented a significant impact of disability in PwP, caregivers’ mood, and caregiving hours on caregiver burden [19,69,70]. Investigating these aspects in further studies can be helpful, especially because therapies for depression, for example, are available and may therefore help to reduce caregiver burden.

### 4.3. Limitations

First, this study is a monocentric, cross-sectional observational study. Therefore, we cannot make any statements about the influence of neuropsychiatric symptoms on HR-QoL of PwP, their caregivers, and caregiver burden over time. Longitudinal data would be desirable to study the dynamics of the investigated factors. Furthermore, this study did not take other aspects, such as already established treatments (medication, occupational therapy) for neuropsychiatric aspects, into consideration. Additionally, neuropsychiatric symptoms are only one part of many non-motor symptoms in PD. They further include troubles like pain, sleep disorders, fatigue, and autonomic dysfunctions, which all have an impact on the patients’ QoL [43,71]. These symptoms might interfere with neuropsychiatric symptoms, but were not investigated in this study. Another important point is that neuropsychiatric symptoms do not only have a direct, but also indirect influence on QoL, as they augment disability and cognitive dysfunction [71,72]. As mentioned before, cognitive impairment was not specifically investigated by a specific test in this study, but should be taken into consideration in future studies.

Even though we used the validated SEND-PD in our study, this self-evaluated scale reports symptoms in a subjective, rather than objective way. Therefore, a more objective approach (e.g., assessing neuropsychiatric symptoms by a physician or molecular imaging) to investigate the association between neuropsychiatric symptoms and HR-QoL, as well as caregiver burden would be interesting in the future. However, the approach with self-evaluated scales, as done in this study, is more uncomplicated and easier to perform in a clinical routine, and is a widely used strategy in the research field of PD. 

Moreover, a selection bias cannot be ruled out, since severely burdened caregivers often do not participate, as reported by former studies of our group [30,73]. Additionally, more interested PwP and their caregivers concerning the topic of the study were more likely to participate. Another restriction is the only moderate number of included PwP and their caregivers.

## 5. Conclusions

To our knowledge, this was the first study using the SEND-PD in a German cohort to investigate the relationship between neuropsychiatric symptoms in PwP and HR-QoL. A significant association between a reduced HR-QoL of PwP and neuropsychiatric symptoms could be detected, and mood/apathy were especially bothersome. Furthermore, mood/apathy correlated significantly with a higher caregiver burden of the informal caregiver measured by a disease-specific questionnaire. Further studies are needed to confirm these findings and to investigate if therapeutic interventions for neuropsychiatric symptoms, such as medication, psychotherapy, and occupational treatment have a positive influence on HR-QoL and caregiver burden in PD [73,74].

## Figures and Tables

**Table 1:** PwP (n = 89; n = 34 females) and caregiver (n = 84; n = 54 females) characteristics.

	Mean ± SD	Median	Minimum	Maximum
PwP				
Age (years)	68.7 ± 10.1		41	88
Disease duration (years)	10.7 ± 6.6		1	29.2
Hoehn and Yahr stage		3.0	1	5
PDQ-8	34.8 ± 17.8		3.1	84.4
MDS-UPDRS part II	17.7 ± 10.6		4	48
BDI	12.0 ± 8.0		0	48
SEND-PD psychotic symptoms	1.3 ± 2.2		0	12
SEND-PD mood/apathy	4.9 ± 4.4		0	20
SEND-PD impulse control disorders	1.1 ± 1.5		0	7
Caregivers				
Age (years)	66.2 ± 10.6		36	88
Caregiving hours per day	5.5 ± 6.5		0	24
PDCB	31.5 ± 16.0		0	79
BDI	9.2 ± 6.8		0	28
SF-36 total	64.0 ± 19.6		16.4	93.0

BDI, Beck depression inventory; MDS-UPDRS, Movement Disorder Society unified Parkinson’s disease rating scale; PDCB, Parkinson’s disease caregiver burden questionnaire; PDQ-8, Parkinson’s disease quality of life questionnaire 8; SEND-PD, Scale for evaluation of neuropsychiatric disorders in Parkinson’s disease; SD, Standard deviation; SF-36, short-form 36 health.

**Table 2:** Spearman correlation of disease-related factors and PwP’s health-related quality of life (PDQ-8, n = 87).

	R	*p*	n
Disease duration in years	0.184	0.117	74
Hoehn and Yahr stage	0.497	**0.000**	83
MDS-UPDRS II	0.487	**0.000**	86
SEND-PD psychotic symptoms	0.341	**0.002**	84
SEND-PD mood/apathy	0.504	**0.000**	87
SEND-PD impulse control disorders	0.323	**0.003**	83
Caregiving hours/day	0.579	**0.000**	77

Spearman correlation of disease-related factors and PwP’s health-related quality of life (PDQ-8). *p* value adjustment for multiple comparisons was 0.05/6 = 0.008. Significance level at *p* < 0.008 are printed in **bold**. MDS-UPDRS II, Movement Disorder Society unified Parkinson’s disease rating scale; n, number of included PwP; SEND-PD, Scale for evaluation of neuropsychiatric disorders in Parkinson’s disease.

**Table 3:** Multiple linear regression analysis of factors contributing to PwP’s HR-QoL (PDQ-8, n = 87); *R* = 0.600; *R*^2^ = 0.360; adjusted *R*^2^ = 0.336.

	B (95% Confidence Interval)	Beta	*t*	*p*
SEND-PD psychotic symptoms	1.920 (−0.1.322; 3.162)	0.101	0.817	0.416
SEND-PD mood/apathy	2.431 (1.305; 3.557)	0.577	4.297	**0.000**
SEND-PD impulse control disorders	−1.398 (−3.959; 1.163)	−0.114	−1.087	1.163

Multiple linear regression analyses of factors contributing to PwP’s HR-QoL. *p* value adjustment for multiple comparisons was 0.05/3 = 0.017. Significance level at *p* < 0.017 is printed in **bold**. SEND-PD, Scale for evaluation of neuropsychiatric disorders in Parkinson’s disease.

**Table 4:** Spearman correlation of disease-related factors and caregiver burden (PDCB, n = 82).

	R	*p*	n
Disease duration in years	0.219	0.072	68
Hoehn and Yahr stage	0.192	0.095	77
MDS-UPDRS II	0.487	**0.000**	80
SEND-PD psychotic symptoms	0.410	**0.000**	82
SEND-PD mood/apathy	0.584	**0.000**	82
SEND-PD impulse control disorders	0.405	**0.000**	81
Caregiving hours/day	0.342	**0.003**	74
Caregivers’ BDI	0.696	**0.000**	82

Spearman correlation of disease-related factors and caregiver burden (PDCB). *p* value adjustment for multiple comparisons was 0.05/8 = 0.006. Significance level at *p* < 0.006 is printed in **bold**. BDI, Beck’s Depression Inventory, MDS-UPDRS II, Movement Disorder Society unified Parkinson’s disease rating scale; n; number of included caregivers; SEND-PD, Scale for evaluation of neuropsychiatric disorders in Parkinson’s disease.

**Table 5:** Multiple linear regression analysis of factors contributing to caregiver burden (PDCB, n = 82); *R* = 0.630; *R*^2^ = 0.397; adjusted *R*^2^ = 0.373.

	B (95% Confidence Interval)	Beta	*t*	*p*
SEND-PD psychotic symptoms	1.187 (−0.474; 2.848)	0.165	1.423	0.159
SEND-PD mood/apathy	1.711 (0.768; 2.653)	0.444	3.615	**0.001**
SEND-PD impulse control disorders	1.338 (−0.884; 3.560)	−0.120	−1.199	0.234

Multiple linear regression analyses of factors contributing to caregiver burden. *p* value adjustment for multiple comparisons was 0.05/3 = 0.017. Significance level at *p* < 0.017 is printed in **bold**. SEND-PD, Scale for evaluation of neuropsychiatric disorders in Parkinson’s disease.

## Data Availability

Data are available on reasonable demand to M.K. as corresponding author.

## Appendix A

**Table A1:** Numbers of PwP and neuropsychiatric symptoms depending on disease duration.

		<5 Years(n = 15)	5–15 Years (n = 37)	>15 Years (n = 19)
SEND-PD psychotic symptoms	Mean ± SD	2.3 ± 0.8	2.3 ± 0.68	2.8 ± 0.4
	n	8	16	12
SEND-PD mood/apathy	Mean ± SD n	4.5 ± 1.1 13	4.8 ± 0.6 31	7.8 ± 1.2 17
SEND-PD impulse control disorders	Mean ± SD n	1.7 ± 0.29 7	2.3 ± 0.5 16	2.4 ± 0.4 14

Numbers of PwP and neuropsychiatric symptoms depending on disease duration. PwP were considered to have a neuropsychiatric symptom when reporting a SEND-PD subscale score ≥ 1. n = number of PwP. SEND-PD, Scale for evaluation of neuropsychiatric disorders in Parkinson’s disease.

**Table A2:** Numbers of PwP and neuropsychiatric symptoms depending on age.

		<50 Years (n = 4)	51–70 Years (n = 39)	>70 Years (n = 39)
SEND-PD psychotic symptoms	Mean ± SD	1.0 ± 0.0	2.4 ± 0.6	2.4 ± 0.5
	n	2	17	21
SEND-PD mood/apathy	Mean ± SD n	5.3 ± 1.5 3	5.2 ± 0.7 35	6.0 ± 0.8 33
SEND-PD impulse control disorders	Mean ± SD n	1.3 ± 0.3 4	2.4 ± 0.4 20	2.0 ± 0.3 19

Numbers of PwP and neuropsychiatric symptoms depending on age. PwP were considered to have a neuropsychiatric symptom when reporting a SEND-PD subscale score ≥ 1. n = number of PwP. SEND-PD, Scale for evaluation of neuropsychiatric disorders in Parkinson’s disease.
